# Radiation Recall in a Patient with Intrahepatic Cholangiocarcinoma: Case Report and a Literature Review

**DOI:** 10.7759/cureus.5020

**Published:** 2019-06-27

**Authors:** Waled Bahaj, Lina Ya'qoub, Muhammad Toor, Ashiq Masood

**Affiliations:** 1 Internal Medicine, University of Missouri-Kansas City School of Medicine, Kansas City, USA; 2 Oncology, University of Missouri-Kansas City School of Medicine, Kansas City, USA

**Keywords:** recall dermatitis, rash, gallbladder cancer

## Abstract

Radiation recall dermatitis (RRD) is a rare and poorly understood phenomenon, constituting an inflammatory skin reaction to a previously irradiated area of skin following the administration of certain agents, usually chemotherapy. Our patient developed RRD 66 years after receiving radiation therapy; to the best of our knowledge, this is the longest reported period in the literature. The mainstay of therapy is to withhold the agent that elicited the adverse reaction, followed by symptomatic management. Subjecting patients to further chemotherapy can provoke another episode of RRD. Therefore, clinical judgment in this regard is usually recommended.

## Introduction

Radiation recall dermatitis (RRD) is a rare acute inflammatory reaction of the skin that is limited to previously irradiated skin after the administration of certain agents [1], usually chemotherapy [2]. However, RRD is associated with other medications, including simvastatin, Tamoxifen, and antibiotics [3-4]. It generally appears months to years after radiation therapy, although some cases have been reported after only a few hours or days [2, 5]. Thus, this phenomenon appears to be independent of the duration between the administration of radiation and the appearance of geographically limited dermatitis. To the best of our knowledge, the longest reported duration between the administration of radiation and drug-induced RRD is 25 years [6]. Here, we present a case of a patient who developed RRD 66 years after receiving radiation therapy. 

## Case presentation

**Figure 1:**
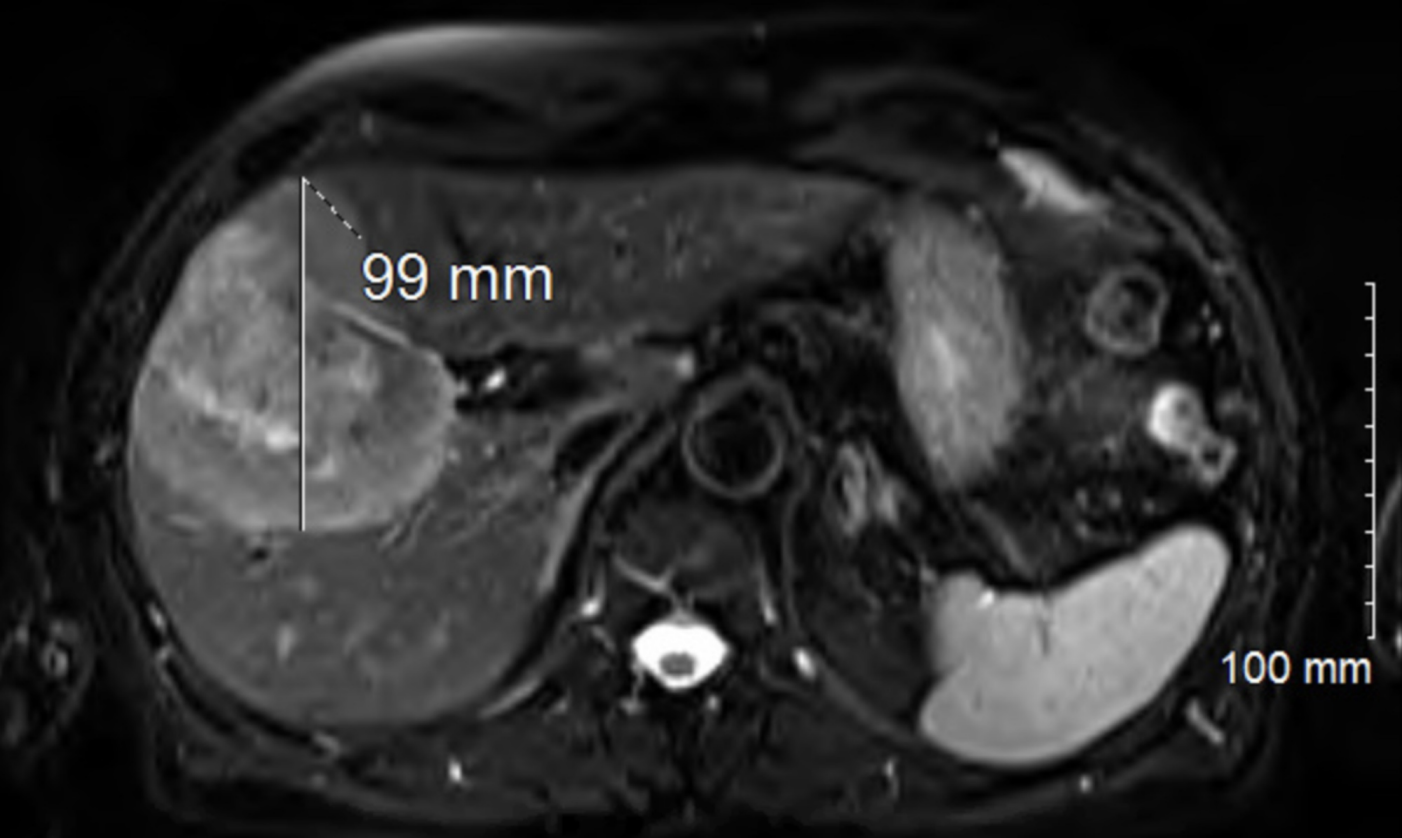
MRI abdomen showing enhancing mass with necrotic center

A 79-year-old man with a history of right arm soft-tissue tumor of unknown etiology and who had been treated with surgical resection followed by radiation therapy (an unknown dose) to the right arm and shoulder area in 1951, presented at our institution in February 2017 with right-upper-quadrant abdominal pain and weight loss of 10 pounds. Subsequently, he underwent an abdominal ultrasound that revealed a large heterogeneous liver mass measuring 8 cm × 5 cm × 7 cm. An enhancing mass with a necrotic center that involved the right liver lobe (segments 5 and 8) and slightly extended into the left hepatic lobe (segment 4), with encasement by at least one portal vein, was demonstrated using magnetic resonance imaging of the abdomen (Figure 1).

**Figure 2:**
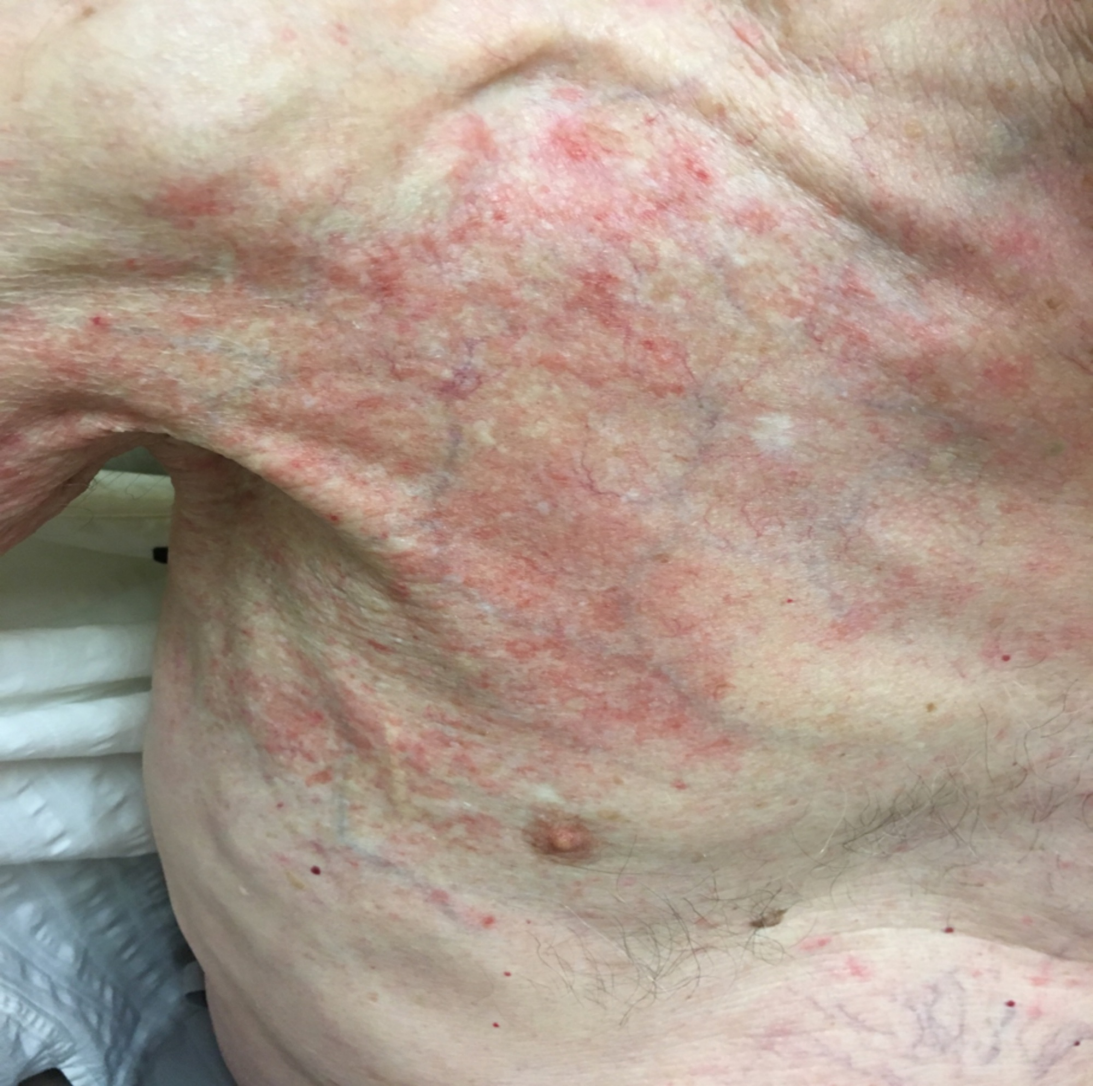
Radiation recall dermatitis in the patient’s right arm and shoulder, extending to the anterior chest wall.

The finding of an ultrasound-guided liver biopsy was consistent with intrahepatic cholangiocarcinoma. After discussions among a multidisciplinary healthcare team, the surgical option was contraindicated. The patient was treated with a combination of simvastatin, tamoxifen (1,000 mg/m^2^) and cisplatin (25 mg/m^2^). Four days after cycle one, day eight of gemcitabine, he developed an erythematous, coalescent patchy rash on the right shoulder and arm that extended into the chest wall and was geographically restricted to the site of previous radiation treatment for a soft-tissue tumor (Figure 2).

The diagnosis was consistent with RRD manifesting 66 years of radiation treatment. He was managed symptomatically with topical triamcinolone and oral antihistamines. The rash improved and completely resolved within three weeks. Upon resolution of dermatitis, the patient was rechallenged with the same dose of gemcitabine/cisplatin, and on this occasion, he tolerated it well.

## Discussion

The pathogenesis of RRD is not fully understood, and different mechanisms have been proposed in this regard. These include local vascular injury with a permeability defect that affects the pharmacokinetics of certain drugs, thereby causing RRD, as well as epithelial stem-cell depletion in the irradiated area, resulting in a recognized reaction in the surviving non-affected cells or mutation in these cells that ultimately led to intolerability of the chemotherapeutic agent. Another suggested theory involves idiosyncratic drug non-immune reactions [7-8]

Cases of RRD that are characterized by a duration of ≥ 1 year between radiation therapy and the institution of systemic chemotherapy are depicted in Table 1. Our patient developed RRD 66 years after receiving radiotherapy, which is by far the longest reported period. The time interval between the administration of the agent and the development of RRD also differs between cases; the longest reported interval is seven years [9] while some patients develop a reaction within hours [5]. In the current case, the patient developed RRD 11 days after the administration of gemcitabine and cisplatin. RRD manifests as a maculopapular rash, desquamation, pruritis, swelling, or ulceration of the skin [10]. As was evidenced in our patient, RRD usually appears after the first exposure to the eliciting drug [2]. Adverse reactions are associated with specific medications, such as anthracyclines, taxanes, and antimetabolites like gemcitabine, capecitabine, and pemetrexed [11]. It should be borne in mind that medication type does not correlate with a prolonged interval between radiation exposure and RRD. All reported cases with an interval of ≥ 1 year were demonstrated to have been triggered by different agents. Similarly, gemcitabine has been associated with RRD that occurred at different time intervals [12].

**Table 1:** Cases of radiation recall dermatitis characterized by a duration of ≥ 1 year between radiation exposure and the institution of systemic chemotherapy 1a: Time between radiation exposure and radiation recall dermatitis 2b: Time between receiving medication and radiation recall dermatitis in a previously irradiated skin area.

Author	Reason for radiation	Radiation region	Chemotherapy type	Duration 1^a ^	Duration 2^b^	Management
Barlési et al., 2006 [6]	Lung adenocarcinoma with breast metastasis	Breast	pemetrexed, prednisone	25 years	3 days	Prednisone (1 mg/kg). Improvement was seen in two days. The resolution achieved in two weeks.
Burdon et al., 1978 [16]	Sarcoma of the palate	Unspecified	doxorubicin, cyclophosphamide, vincristine sulfate, and high-dose methotrexate with folinic acid rescue	15 years	2 weeks	Drugs were withheld. Topical amphotericin B and nystatin were applied. Stomatitis resolved in seven weeks.
Taunk et al., 2011 [17]	Early-stage breast cancer	Whole left breast	rosuvastatin, amlodipine	5 years	2 weeks	Moisturizing lotion was placed on the affected area, together with 1% hydrocortisone cream. The skin reaction disappeared at 21 days
Parry et al., 1992 [18]	Breast cancer	Breast area	tamoxifen	2 years	5 days	Resolved in two weeks. The patient was rechallenged with a smaller dose. A mild rash resulted.
Bokemeyer et al., 1996 [19]	Advanced breast cancer	Left breast and left chest wall	paclitaxel	2 years	5 days	Chemotherapy was discontinued.
Marisavljevic et al., 2005 [20]	Hodgkin’s lymphoma		gemcitabine, decadrone	2 years	2 days	None. The rash resolved in 10 days. There was a mild recurrence of it after each the administration of gemcitabine.
Abadir et al., 1995 [3]	Carcinoma of the gall bladder	Unspecified	simvastatin	1 year	2-3 days	Unspecified.

Our patient also received cisplatin. Upon reviewing the literature, it was ascertained that only one case of cisplatin-associated RRD has been reported. In that particular case, the patient was started on gemcitabine at the same time. However, in our opinion, it is likely that the adverse reaction was caused by gemcitabine as the latter has been linked to a greater number of RRD cases compared to cisplatin [13]. It is also more probable that high doses of radiation cause RRD. Stelzer et al. evaluated the impact of three different radiation doses (i.e., 8 Gy, 20 Gy, and 40 Gy) on different Kaposi sarcoma areas in one patient in their study. Bleomycin was seen to cause RRD, but this was only evident in the area that received 40 Gy [14].

We successfully rechallenged our patient; he received the second cycle of the same dose of gemcitabine/cisplatin after one month of RRD and tolerated it very well. It is not been elucidated whether or not it is acceptable to rechallenge a patient with another cycle of chemotherapy after RRD. Most clinicians tend to avoid doing this to prevent a recurrence. Only a handful of cases have previously been successfully rechallenged, and this has depended on the treatment approach used. Some clinicians prefer to pretreat patients with steroids, while others opt for a dose reduction, mild cases, as well as cases without recurrence, have been reported [6].

The symptomatic management of RRD is the recommended approach. Our patient experienced immediate relief with the use of triamcinolone cream and diphenhydramine while in the clinic. His rash resolved completely within three weeks of the addition of cetirizine. It is also recommended that the causative agent should be withheld or discontinued until healing takes place. Topical or systemic steroids, nonsteroidal anti-inflammatory medications, and antihistamines have been used for symptomatic relief, and healing is often achieved within two weeks [1-2]. Surgical intervention with wound excision and debridement is the last resort if complicated reactions persist despite the proposed management [9].

Radiosensitization is more common than RRD. It is important to distinguish between these two entities, and this can be achieved by considering the timing. Camidge et al. suggest that an adverse skin reaction to radiation with the simultaneous administration of chemotherapy, and/or within a seven-day timeframe, should be viewed as radiosensitization rather than RRD [2]. Interestingly, trials in which the administration of chemotherapy was deferred for a few days following radiation exposure resulted in a dramatic reduction in the skin reaction [15].

## Conclusions

In summary, following the exposure to the provoking drug, RRD usually appears in a previously irradiated skin area. Our case highlights that RRD can occur decades after initial exposure to radiation and clinicians should be aware of this when treating patients presenting with skin changes in the same geographic area of radiation. Discontinuation of the offending agent and symptomatic management are the recommended approaches of treating RRD.
